# International medical graduates in family medicine in the United States of America: an exploration of professional characteristics and attitudes

**DOI:** 10.1186/1478-4491-4-17

**Published:** 2006-07-18

**Authors:** Amanda L Morris, Robert L Phillips, George E Fryer, Larry A Green, Fitzhugh Mullan

**Affiliations:** 1 Sigma Medical Group, West Lafayette, Indiana, USA; 2 Robert Graham Center Policy Studies in Family Medicine and Primary Care, Washington, DC, USA; 3 Department of Pediatrics, New York University, New York City, USA; 4 Department of Health Policy, George Washington University School of Public Health and Health Services, Washington, DC, USA

## Abstract

**Background:**

The number of international medical graduates (IMGs) entering family medicine in the United States of America has steadily increased since 1997. Previous research has examined practice locations of these IMGs and their role in providing care to underserved populations. To our knowledge, research does not exist comparing professional profiles, credentials and attitudes among IMG and United States medical graduate (USMG) family physicians in the United States. The objective of this study is to determine, at the time when a large influx of IMGs into family medicine began, whether differences existed between USMG and IMG family physicians in regard to personal and professional characteristics and attitudes that may have implications for the health care system resulting from the increasing numbers of IMGs in family medicine in the United States.

**Methods:**

This is a secondary data analysis of the 1996–1997 Community Tracking Study (CTS) Physician Survey comparing 2360 United States medical graduates and 366 international medical graduates who were nonfederal allopathic or osteopathic family physicians providing direct patient care for at least 20 hours per week.

**Results:**

Compared to USMGs, IMGs were older (p < 0.001) and practised in smaller (p = 0.0072) and younger practices (p < 0.001). Significantly more IMGs practised in metropolitan areas versus rural areas (p = 0.0454). More IMG practices were open to all new Medicaid (p = 0.018) and Medicare (p = 0.0451) patients, and a greater percentage of their revenue was derived from these patients (p = 0.0020 and p = 0.0310). Fewer IMGs were board-certified (p < 0.001). More IMGs were dissatisfied with their overall careers (p = 0.0190). IMGs and USMGs did not differ in terms of self-rated ability to deliver high-quality care to their patients (p = 0.4626). For several of the clinical vignettes, IMGs were more likely to order tests, refer patients to specialists or require office visits than USMGs.

**Conclusion:**

There are significant differences between IMG and USMG family physicians' professional profiles and attitudes. These differences from 1997 merit further exploration and possible follow-up, given the increased proportion of family physicians who are IMGs in the United States.

## Background

International Medical Graduates (IMGs) comprised 20.8% of the family physician workforce in 1995 [1]. In 2003, this number increased to 22.1% and is expected to continue to rise, given the large increase of IMGs entering family medicine residency programmes [1].

Historically, family medicine has a smaller proportion of IMGs entering residency training than other primary-care specialties. This relationship is changing as IMGs increasingly filled family medicine residency positions not selected by United States Medical Graduates (USMGs), particularly those vacant after the annual Match [2].

In the 2005 Match, 36.5% of PGY-1 positions available in family medicine residencies were filled by IMGs, an increase of 97 positions from the previous year [3]. On 1 July 2005, IMGs comprised 39.6% of first-year family medicine residents, compared to 14.7% in 1998 [3]. This rise is unique to family medicine, as the number of IMGs entering other primary-care fields has remained relatively stable over the past eight years [4].

Earlier research documented that certain residency programmes have become "dependent" on IMGs, who occupy most or all of their positions [2,5]. It is possible that differences in training backgrounds may translate to important differences in the practice of medicine relating to issues such as health care costs and access to care. As the United States relies heavily on family physicians to serve a large portion of its population [6], it is important to consider what, if anything, this influx of IMGs might portend for the future of the specialty and health care in the nation.

IMG's geographical and specialty distributions, their contribution to meeting the needs of the medically underserved, and the value of Conrad-20 and other visa-waiver programmes that integrate them into the United States physician workforce have been examined in past studies [7-10]. A few studies have focused on IMGs performance at the residency training level and their post-training medical practices [11-13]. Although several of these studies evaluate IMGs in primary-care specialties, none of them focus solely on the field of family medicine. Previous research has neither described the attributes and influences of IMG medical practices nor characterized IMGs' professional credentials and attitudes beyond the residency level in family medicine.

In this paper we examine personal, professional and practice characteristics and attitudes of IMG and USMG family physicians in order to provide insight into practice differences that may affect the health care system. We use data from a nationally representative survey collected in 1996–1997, a time when major increases in the proportion of IMGs in family medicine residency training began, to answer the question: "Do significant differences exist between IMG and USMG family physicians that warrant further investigation?"

We compare physician-reported personal, professional and practice characteristics, attitudes regarding clinical care, assessments of various influences on the delivery of medical care, referral patterns and responses to clinical vignettes between the two groups. Beyond forming a baseline comparison and characterization for future analyses, this analysis highlights findings that may have implications for the health care system as a whole resulting from the increasing numbers of IMGs in family medicine in the United States.

## Methods

### Data sources

Data used in this study were collected through the Community Tracking Study (CTS) Physician Survey, a project of the Center for Studying Health System Change, in Washington, DC, and obtained from the Inter-University Consortium for Political and Social Research (ICPSR), in Ann Arbor, Michigan [14]. The CTS is a biannual survey sponsored by the Robert Wood Johnson Foundation to study changes in the health care system and their impact on individuals. The sample frame consisted of nonfederal MDs and DOs who provided direct patient care for at least 20 hours per week.

The 1996–1997 CTS is the most recent source of a nationally representative sample containing special provisions to examine practice characteristics and tendencies in addition to the responses to 12 clinical vignettes. Primary-care physicians were oversampled, and of the 2726 family physicians who participated, 2360 were USMGs and 366 were IMGs. Residents and fellows were not surveyed. The overall response rate was 65% and the response number to each individual item is listed in each table. The CTS does not identify the specific response rates between IMG and USMG participants.

### Study variables

We compared responses to survey items between IMG and USMG family physicians. The CTS defined International Medical Graduates as graduates of medical schools outside the United States, Canada and Puerto Rico. The CTS also included the responses from general practitioners in the family physician group.

We examined several survey items relating to personal and professional characteristics including age, years in practice, net income and board-certification status. We assessed various practice characteristics including practice location (large metro, small metro, non-metro as defined by the 1995 United States Census), practice type (solo or two physicians, group practice, health maintenance organization (HMO), hospital-based, medical school, other), net income and percentages of income from various sources (Medicare, Medicaid, HMO, bonuses). We also compared the number of new Medicare and Medicaid patients each physician was accepting into his or her practice.

The survey items queried physicians' agreement with statements regarding clinical care using a five-point Likert scale ("disagree strongly", "disagree somewhat", "neither agree nor disagree"," agree somewhat", or "agree strongly"). There were few "neither agree nor disagree" responses. In order to compare each group, we eliminated the category and collapsed the remaining groups into two response groups: "agree" or "disagree", as has been done with analysis of these data in other research [15].

The physicians were similarly asked about career satisfaction ("Thinking very generally about your overall career in medicine, would you say that you are currently very satisfied, somewhat satisfied, somewhat dissatisfied, very dissatisfied, or neither satisfied or dissatisfied?"). These responses were collapsed to "dissatisfied" or "satisfied" for our analysis.

We compared survey items that assessed various influences on the physicians' practice of medicine. These items include the effects of using computers to obtain patient data and treatment guidelines, using preventive treatment reminders and formal written guidelines, and the effects of practice profile results and patient satisfaction surveys.

In order to compare referral patterns among physicians, we analysed responses to survey items regarding the number of patients the physicians referred to specialists, the complexity of the medical conditions of those patients they did not refer to specialists, and the complexity of medical conditions the physicians felt specialists expected them to care for without referral. These survey items asked each physician to state whether each item had "decreased a lot", "decreased a little", "stayed the same", "increased a little", or "increased a lot" over the previous two years.

The 1996–1997 CTS contained 12 clinical vignettes that queried physicians about medications, tests and referrals they would order for various clinical scenarios.

### Analytic strategy

USMG and IMG practice characteristics, professional attitudes and satisfaction were contrasted by means of chi-square and t-tests. SUDAAN software, version 8.0 (Research Triangle Institute, Research Triangle Park, North Carolina, United States of America) was used for statistical comparisons and making national estimates. SUDAAN adjusted variance due to non-response and CTS survey design complexities. Statistical significance was set at p-values less than 0.05.

## Results

Of the 2726 family physicians surveyed, 2360 were USMGs and 366 were IMGs. We found that IMGs were older than USMGs, but significantly more likely to have been in practice for fewer years (Table 1). IMGs were more likely to practise in metropolitan areas than in non-metropolitan areas, with 82% of IMGs in metropolitan areas compared to 75% of USMGs (p = 0.0454). A greater percentage of IMGs were full owners of their practices and more often in solo or two-physician groups. Only 67% of IMGs were board-certified, compared to 87% of USMGs (p < 0.001). More IMGs were accepting all new Medicare (67% versus 60%, p = 0.0451) and Medicaid (49% versus 40%, p = 0.0018) patients than USMGs. They also derived a greater percentage of their revenue from Medicare and Medicaid than their USMG counterparts. IMGs served as "gatekeepers" and were required to provide permission to see specialists for a greater percentage of their patients than USMGs. Differences between IMGs and USMGs regarding net income, hours in direct patient care, percentage of revenue from managed care, and percentage of income from bonuses were not significant (data not shown).

**Table 1:** Personal, professional and practice characteristics of IMG and USMG family physicians

	**USMGs **N = 2360 n (%)*	**IMGs **N = 366 n(%)*	p value**
**Age **(n = 2591)			
<35	299 (13)	13 (3)	**<0.001**
35–44	949 (43)	106 (33)	
45–54	588 (26)	117 (33)	
55–64	251 (12)	63 (18)	
>65	163 (7)	42 (13)	
**Years in practice **(n = 2726)			
Under 6	449 (19)	81 (23)	**<0.001**
6–10	504 (22)	51 (12)	
11–20	830 (35)	121 (34)	
21–30	279 (12)	56 (15)	
31–40	234 (10)	43 (12)	
41+	64 (3)	14 (4)	
**Location **(n = 2726)			
Large metro >200 K	1955 (70)	322 (76)	**0.0454**
Small metro <200 K	45 (5)	7 (6)	
Non-metro area	360 (25)	37 (18)	
**Ownership status **(n = 2726)			
Full owner	733 (32)	150 (43)	**<0.001**
Part owner	472 (21)	52 (14)	
Not an owner	1155 (48)	164 (43)	
**Practice type **(n = 2726)			
Solo or 2 physicians	829 (37)	169 (47)	**0.0072**
Group ≥ 3 physicians	585 (24)	76 (21)	
Health maintenance organization	154 (5)	27 (5)	
Medical school	89 (4)	11 (4)	
Hospital-based	388 (17)	46 (13)	
Other	315 (13)	37 (9)	
**Board certification status **(n = 2707)			
Board Certified	2055 (87)	243 (67)	**<0.001**
Board Eligible Only****	169 (7)	61 (18)	
Neither	123 (6)	56 (15)	
**Accepting new Medicare patients **(n = 2726)			
None	222 (9)	27 (9)	**0.0451**
Some	418 (17)	43 (12)	
Most	339 (14)	51 (12)	
All	1381 (60)	245 (67)	
**Accepting new Medicaid patients **(n = 2726)			
None	647 (25)	94 (24)	**0.0018**
Some	591 (25)	67 (18)	
Most	231 (10)	35 (9)	
All	891 (40)	170 (49)	
**Percentage of revenue from Medicare **(n = 2726)	Mean (SE) 27.54 (0.48)	Mean (SE) 30.62 (1.46)	0.0310***
**Percentage of revenue from Medicaid **(n = 2726)	Mean (SE) 13.90 (0.48)	Mean (SE) 17.82 (1.31)	0.0020***
**Percentage of patients for whom physician serves as gatekeeper **(n = 2726)	Mean (SE) 36.72 (0.94)	Mean (SE) 43.67 (2.34)	0.0018***

*Unweighted number of survey respondents and weighted percentage of family physicians. Numbers vary because not all survey items were answered by each physician.

** Chi-Square

*** T-Test

****Although "Board Eligible" is not a status officially recognized by the ABFM, it is one of the responses offered by the CTS.

USMG = United States Medical Graduate, IMG = International Medical Graduate, SE = Standard Error

IMGs were more likely to report having adequate time to spend with patients (73% versus 66%, p = 0.0041) and being able to develop continuing patient relationships (83% versus 78%, p = 0.0096) than USMGs, yet more IMGs were dissatisfied with their overall medical careers (20% versus 16%, p = 0.0190) than USMGs (Table 2).

**Table 2:** Attitudes regarding clinical care of IMG and USMG family physicians

	**USMGs **N = 2360 n (%)*	**IMGs **N = 366 n(%)*	p value**
**"I have adequate time to spend with my patients during typical office/patient visits." **(n = 2664)			
Disagree	813 (34)	102 (27)	**0.0041**
Agree	1492 (66)	257 (73)	
**"It is possible to maintain the kind of continuing relationships with patients over time that promote the delivery of high quality care." **(n = 2651)			
Disagree	559 (22)	63 (17)	**0.0096**
Agree	1738 (78)	291 (83)	
**"It is possible to provide high-quality care to all my patients." **(n = 2679)			
Disagree	442 (19)	73 (18)	0.4626
Agree	1875 (81)	289 (82)	
**"I have the freedom to make clinical decisions that meet my patients' needs." **(n = 2685)			
Disagree	332 (14)	61 (17)	0.1677
Agree	1991 (86)	301 (83)	
**"The level of communication I have with specialists about the patients I refer to them is sufficient to ensure the delivery of a high quality of care." **(n = 2679)			
Disagree	276 (11)	46 (12)	0.5777
Agree	2045 (89)	312 (88)	
**"I can make clinical decisions in the best interests of my patients without the possibility of reducing my income." **(n = 2601)			
Disagree	447 (19)	74 (21)	0.5571
Agree	1800 (81)	280 (79)	
**Overall career satisfaction **(n = 2678)			
Dissatisfied	382 (16)	79 (20)	**0.0190**
Satisfied	1978 (84)	287 (80)	

*Unweighted number of survey respondents and weighted percentage of family physicians. Numbers vary because not all survey items were answered by each physician.

** T-Test

USMG = United States Medical Graduate, IMG = International Medical Graduate

There were no significant differences between IMGs and USMGs regarding perceived ability or freedom to provide high-quality care, to communicate with specialists and to make clinical decisions without reducing income. IMGs reported that preventive treatment reminders, formal written guidelines and patient satisfaction surveys had more influences on their practice of medicine than USMGs (data not shown).

There were no significant differences between IMGs and USMGs on the effects of using computers on obtaining patient data and treatment guidelines and the effects of practice profile results (data not shown). Small but significant differences existed between IMGs and USMGs with regard to referral patterns, but no meaningful trends were identified (data not shown).

The responses to the 12 clinical vignettes are listed in Table 3. For many clinical scenarios, IMGs were more likely to order tests, refer patients to specialists or require office visits than USMGs.

**Table 3:** Responses to clinical vignettes of IMG and USMG family physicians

**Clinical vignette**	**USMGs N = 2360 Mean (SE)**	**IMGs N = 366 Mean (SE)**	p value*
% 50 y/o males prescribed oral agents for total cholesterol of 240, LDL of 150 and HDL of 50 after 6 months of diet therapy and no other cardiac risk factors (n = 2726)	21.60 (0.87)	22.92 (1.71)	0.4533
% 60 y/o males referred to urology for moderate BPH symptoms without signs of renal disease or cancer (n = 2726)	11.65 (0.55)	15.52 (1.77)	0.0276
% 50 y/o males with 1 month history of exertional chest pain referred to Cardiology after an abnormal stress test (n = 2726)	47.20 (1.15)	50.93 (2.80)	0.1791
% 35 y/o males ordered MRI for acute low back pain with new left foot drop (n = 2726)	33.39 (0.90)	39.58 (1.97)	0.0036
% Asymptomatic, male, Caucasian patients > 60 y/o without family history of prostate cancer and normal digital rectal exams who are ordered PSA test (n = 2726)	37.31 (1.34)	42.57 (2.64)	0.0301
% 40 y/o monogamous, female patients with 2 days of vaginal itching and thick white discharge without abdominal pain or fever who are required an office visit (n = 2726)	21.84 (0.95)	34.91 (2.61)	<0.001
% 10 y/o males with primary enuresis with negative work-up and failure of water restriction and environmental measures who are prescribed DDAVP (n = 2726)	26.23 (0.84)	31.98 (2.50)	0.0265
% 10 y/o males with 2 day history of sore throat and fever who required an office visit (n = 2726)	31.74 (1.03)	25.92 (1.99)	0.0084
% 10 y/o females with fever, tachypnea and right-basilar rales who are ordered chest X-rays (n = 2726)	23.40 (1.01)	27.56 (2.22)	0.0574
% 24-month-old females with history of 6 resolved episodes of supporative otitis media in past year with normal hearing and failure of prophylactic antibiotics who are referred to ENT for PE tubes (n = 2726)	22.94 (0.87)	22.60 (1.97)	0.8816
% Full-term, 6-week-old children given sepsis work-up (CBC, sterile urine and blood cultures) for a fever of 101 (n = 2726)	29.54 (0.91)	26.63 (2.25)	0.2458
% 4 y/o children with eczema and asthma that is worsening despite inhaled corticosteroids who are referred to an allergist (n = 2726)	29.42 (0.97)	28.40 (2.31)	0.6605

* T-Test

USMG = United States Medical Graduate, IMG = International Medical Graduate, SE = Standard Error

## Discussion

This study suggests that IMGs and USMGs who practise family medicine differ in important ways. Professionally, fewer IMGs are board-certified, compared to USMGs. Their practices differ from those of USMGs in terms of practice location and service of the Medicare and Medicaid populations. Significantly fewer IMGs report being satisfied overall with their medical careers than USMGs. Furthermore, subtle differences exist between the groups in regards to patient referral patterns and in their responses to several clinical vignettes. These differences, if they continue to exist, may affect important aspects of the health care system, particularly access to care and health care use and costs.

### Fewer IMGs are board-certified than USMGs

The CTS survey data do not allow us to ascertain the etiology of this profound difference. Prerequisites for becoming board-certified in family medicine include unlimited licensure to practise medicine, completion of a three-year residency programme in family medicine, with the last two years being at the same location, and a passing score on the American Board of Family Practice Board Certification Exam.

Physicians surveyed in this study were practising medicine prior to the implementation of the Clinical Skills Assessment (CSA) requirement for IMGs to enter United States residency programmes. This requirement was implemented by the Educational Commission for Foreign Medical Graduates (ECFMG) in July 1998 to evaluate IMGs' clinical and communication skills [16]. Since the implementation of the CSA, the United States Medical Licensing Exam (USMLE) Step 1 and 2 passage rates of IMGs receiving ECFMG certification have increased. We may also see increases in the percentage of board-certified IMG family physicians in the future with this new requirement.

It is uncertain how board certification and exam-passing rates correlate to quality outcomes in clinical practice. An extensive literature review performed in 1997 revealed that insufficient evidence existed to support or refute the use of board certification as a proxy for physician quality [12]. More recent studies suggest that board certification and maintenance of certification may be linked to improved clinical outcomes [17]. Furthermore, the public places a high value on board certification and would potentially change behaviour to ensure their physician is board-certified[17]. The difference between IMGs' and USMGs' board certification status is important, as more emphasis is being placed on this credential. It is also important to address the proposed link between board-certification status and physician quality in future studies.

### Significantly more IMG family physicians are practising in more urban areas as opposed to rural areas

Previous studies found that IMGs comprise a larger portion of the primary-care physician workforce in rural areas with physician shortages than USMGs, but these studies lumped primary care as a single workforce [9,10]. Our study and another national study both reveal that IMGs in family medicine, the primary-care specialty most likely to distribute like the United States population, are less likely to practise in rural areas than USMGs [8].

Although a greater percentage of new physicians entering Health Professional Shortage Areas (HPSAs) are IMGs, the majority of these physicians are temporary visa holders [18]. It is uncertain if this commitment to the underserved is long-term. Furthermore, previous research has revealed that IMGs are more likely to practise in markets with higher concentrations of established IMG physicians [19]. If this trend continues, one may expect IMGs to continue to locate in urban versus rural practices.

The CTS physician survey did not identify HPSAs or visa status, so we were unable to assess the relative placement of IMGs and USMG family physicians in regard to treating the underserved, or their long-term commitment to serving these areas. Additionally, the CTS data do not allow us to perform a more refined analysis of exactly where in urban areas these physicians are located, as other studies looking at IMGs of all specialties have done [7,20].

### More IMG practices are open to all new Medicaid and Medicare patients, and a greater percentage of their revenue is derived from these patients

This trend has also been established in comparing IMGs and USMGs in psychiatry [21]. The greater service of Medicare and Medicaid populations by IMG family physicians suggests they have a greater dependence on publicly financed programmes and that they plan an important role in providing access to care for Americans covered by these federal programmes.

### More IMGs are dissatisfied with their overall medical careers

Previous studies have linked career dissatisfaction with a perceived inability to provide high-quality care [15]. Although more IMGs in this study are dissatisfied than USMGs, they report no less ability to deliver high-quality care to their patients. Furthermore, more IMGs report having adequate time and ability to develop continuing relationships with their patients than their USMG colleagues. Given the link between career satisfaction and quality of care, it is important to consider what potentially increasing the percentage of dissatisfied physicians means for the future of family medicine and the patients they serve. While it is beyond the scope of this study, it is important to further examine this difference in order to discern potential etiologies and solutions to improve physician satisfaction.

In reviewing the clinical vignettes, we noted several situations in which IMGs order more tests, refer more patients or require more office visits than USMGs. These differences were statistically significant for half of the vignettes, but it is uncertain whether this reflects appropriate care or if the differences were clinically significant. To our knowledge, no research has been published examining the referral patterns and service use practices of IMGs and USMG physicians in the United States. This area deserves further examination, as there may be important health care cost and utilization effects to consider if differences between the groups do exist.

As previously stated, it is important to note that there are no significant differences between IMGs and USMGs regarding their self-rated ability to deliver high quality care to their patients. A greater percentage of IMGs feel that they have adequate time to spend with their patients and that they are able to develop continuing relationships with them.

There are several limitations to our study. The cross-sectional nature of this study precludes our ability to demonstrate causal relationships between variables in addition to the potential for selection effects and the inability to assess possible maturation effects. Reporting error and recall bias are always potentials when examining survey data. Furthermore, the data in this study are subjective reports given by physicians themselves, as opposed to objective practice analyses and clinical outcomes.

We are also unable to differentiate between IMGs who were born in the United States and those who were foreign-born. Since United States-born IMGs and "Fifth Pathway" students account for increasing numbers of PGY-1 family practice residents [3], it would be important to determine whether differences exist between this group and their foreign-born counterparts.

The linkage of general practitioners with family physicians by the CTS may affect some of the results of this paper. Although we know the participants were self-reported family physicians or general practitioners, we do not know the primary focus of their practices (traditional practice, emergency physician, occupational medicine, etc.).

In addition, the clinical vignettes were designed by the CTS and their validity is untested. We include them because they offer a unique viewpoint into clinical decision-making that is often not available in data sources.

Our data were collected in 1996 and 1997. This is the most recent nationally representative data source that oversampled primary-care physicians and examined the specific clinical information we needed, including the clinical vignettes. Although the CTS has collected physician survey data in subsequent years (1998–1999, 2000–2001), neither of these data sets include the pertinent clinical information provided in the vignettes of the 1996–1997 data set. In addition, this time frame allows us to obtain a snapshot of family practice physicians at the time when USMG interest in family medicine was peaking and the number of IMGs entering the field was poised to increase, thereby providing a baseline with which to compare more recent studies on this issue.

IMGs were just 13.4% of the CTS sample – less than their percentage in the overall family medicine workforce. We cannot know whether there was a disproportionately lower response rate from IMGs or if IMGs were a smaller proportion of the workforce within the sampled areas. While this may affect external validity, we don't anticipate that non-respondent IMGs would be more satisfied, or less likely to serve Medicare and Medicaid patients. The characteristics reported in this study may have changed over the past nine years.

## Conclusion

At a time when family medicine began a new trend of becoming more populated with IMGs, we found important differences between the professional and clinical attributes and attitudes of IMG and USMG family physicians that may affect the health care system. Unlike previous studies, the practice locations of the IMG family physicians in this study were more urban than those of their USMG peers. IMG family physicians tend to own their practices more often, practise in smaller groups and be more open to serving Medicare and Medicaid patients than their United States-trained colleagues. The lower rate of board certification among IMG physicians is important to consider as more emphasis is being placed on this credential. There were small but significant differences in career satisfaction and referral patterns that are worth exploring both to understand whether they continue to be true, and to understand if they may change the scope and practice of family medicine. Given that the United States relies on family physicians to serve a large portion of its population and the persistent lack of interest in family medicine by United States medical students, we need to further understand these differences and how they relate to the health care system as a whole.

## Competing interests

This study was funded by the Robert Graham Center. The information and opinions contained in research from the Graham Center do not necessarily reflect the views or policies of the AAFP. The authors declare they have no competing interests.

## Authors' contributions

ALM participated in study design, data collection and manuscript development and revision. RLP participated in study design and manuscript development and revision. FM participated in critical revision of the manuscript. LAG participated in study design and manuscript development and revision. GEF participated in study design and data collection and performed the statistical analysis. All authors read and approved the final manuscript.
